# Guernsey’s Bœnninghausen

**Published:** 1889-09

**Authors:** 


### Guernsey's Bœnninghausen.

The celebrated *Therapeutic Pocket-Book of Bœnninghausen* has long been out of print, and it has, therefore, been difficult for one to secure a copy. Dr. William Jefferson Guernsey now publishes a new edition in a novel form. Each symptom is numbered, and is printed upon a long slip of paper; a printed index accompanies these slips, so one can readily find the slips containing any desired symptoms. These slips can then be laid side by side for comparison. There are over two thousand four hundred of these slips, very neatly printed and carefully arranged in a strong card-board box.

This method of using a repertory has been tried by many and has been found a useful one. The work was evidently very laborious, and has cost heavily. The edition is sold only by subscription, at $10.00 per copy. All orders must be sent to Dr. Guernsey, 4430 Frankford Avenue, Philadelphia.

